# The Critical Trigger for Cognitive Penetration: Cognitive Processing Priority over Perceptual Processing

**DOI:** 10.3390/bs14080632

**Published:** 2024-07-24

**Authors:** Jiejie Liao, Yidong Yang, Zhili Han, Lei Mo

**Affiliations:** 1 Key Laboratory of Brain, Cognition and Education Sciences, Ministry of Education, South China Normal University, Guangzhou 510630, China; 2 Institute of Cognitive Sciences Marc Jeannerod CNRS, UMR 5229, 69675 Bron, France; 3 Shanghai Key Laboratory of Brain Functional Genomics (Ministry of Education), School of Psychology and Cognitive Science, East China Normal University, Shanghai 200062, China; 4 NYU-ECNU Institute of Brain and Cognitive Science at NYU Shanghai, Shanghai 200062, China; 5 School of International Studies, NingboTech University, Ningbo 315000, China

**Keywords:** cognitive penetration, size judgment, value information, cognition, perception

## Abstract

The visual perception system of humans is susceptible to cognitive influence, which implies the existence of cognitive perception. However, the specifical trigger for cognitive penetration is still a matter of controversy. The current study proposed that the cognitive processing priority over perceptual processing might be critical for inducing cognitive penetration. We tested this hypothesis by manipulating the processing priority between cognition and perception across three experiments where participants were asked to complete a size-judging task under different competing conditions between cognition and perception. To sum up, we proved that the cognitive processing priority over perceptual processing is critical for cognitive penetration. This study provided empirical evidence for the critical trigger for cognitive penetration.

## 1. Introduction

As the saying goes, “beauty is in the eye of the beholder”. This means that different people have different views on what is beautiful. It is a typical cognitive penetration which refers to the phenomenon that occurs when perception is subject to higher-level cognitive influences like affection [1,2], motivations [3], theoretical presuppositions [4], or linguistic representations [5]. In other words, what a person believes, desires, intends, etc., may alter what one sees, hears, etc.

For the proposal that sensory perception is cognitively penetrable, it is currently a matter of debate as to what triggers such a phenomenon to take place, specifically, under which circumstances it is expected to be observed.

So far, there has been a substantial increase in the arguments describing the mechanisms underlying the occurrence of cognitive penetration. On the one hand, there are some philosophical and psychological theories explaining this phenomenon. For example, Macpherson [6] proposed a two-stage and indirect mechanism to explain the occurrence of cognitive penetration. In their proposed two-stage mechanism, firstly, penetrating beliefs cause non-perceptual character. Secondly, that character would initiate the perceptual character that exists in humans. On the other hand, empirical explanations for cognitive penetration abound. For example, the study by Radel and Clément-Guillotin [7] proved that cognition exerted a top-down influence via motivational factors. Lupyan et al. [8] provided evidence that conceptual information can penetrate early visual processing. They revealed that cognitive penetration derives from category learning rather than simply a bias of perceptual system. Another experiment from Bruner and Postman [2] associated positive, negative, and neutral symbols with different discs. The result turned out to be an overestimation for positive or negative associations, but not for the neutral one. The authors explained that salient visual input would appear to be larger for human beings. Conclusively, no agreement has been reached regarding the mechanism underlying the occurrence of cognitive penetration.

Meanwhile, documented works against the existence of cognitive penetration cannot be ignored. For example, in the arguments of Firestone and Scholl [6], they pointed out several pitfalls that previous cognitive penetration demonstrations were caught in. Because most studies committed one or more of these mistakes, they held that none of the evidence for top-down influence on perception or the alleged cognitive penetration was compelling enough. In addition, some early works opposing cognitive penetrability held that the process for visual stimuli was cognitively impermeable, and top-down influence only biased the decision-making process [9,10]. The work of Klein et al. [11] failed to replicate the findings from Bruner and Postman [2]. They argued that distortions in size perception were merely derived from the method used to ask for size judgments, rather than value cognition. In a recent review on cognitive penetration, the authors stated that those works against the existence of cognitive penetrability emphasized the distinction between cognition and perception. This is the classical modular understanding of perception and cognition, according to which perception is an automatic modular system independent of conscious control [4].

It can be found that previous debates about the existence and mechanism of cognitive penetration mainly focused on the distinguishing boundary between cognition and perception. Considering the long-standing controversy of the occurrence of cognitive penetration, the current research aimed to provide empirical evidence for cognitive penetration from the perspective that the cognitive processing priority over perceptual processing is critical for cognitive penetration. We made this proposal because we found that inconsistent findings on the occurrence of cognitive penetration were related to processing priority between cognition and perception being differently manipulated by researchers. For example, in a work typically proving the existence of cognitive penetration, researchers presented participants with words close to the threshold of conscious perception. The results suggested that participants who had fasted were more likely than satiated participants to perceive masked food-related words [7]. In contrast, studies demonstrating that cognition does not influence perception manipulated the two aspects in another way. One central phenomenon offered in support of impenetrability is the Muller-Lyer illusion. That is, even if we know that the two arrows have the same length, we continue to perceive one as being shorter than the other [12]. Therefore, it can be noted that studies showing cognitive penetration deployed ambiguous stimuli during perception decision, leading to cognitive priority over perception [1], whereas those studies directly presenting stimuli during perception failed to demonstrate cognitive penetration. From the distinct manners of manipulating the relative processing priority of cognition and perception, it can be inferred that cognitive priority over perception may be a critical factor for triggering the occurrence of cognitive penetration. Thus, this study provided empirical evidence for the critical trigger for cognitive penetration. The work of Radel and Clément-Guillotin [7], which used ambiguous experimental stimuli to induce cognitive penetration, concluded that humans interpreted unclear stimuli based on their willingness and desires. This finding was consistent with a consequent proposal from Dunning and Balcetis [5]. Their review held that people categorized ambiguous information from their surroundings in a manner that was in line with their desires.

Considering these debates on the occurrence of cognitive penetration, we assumed that the ambiguous stimuli which led to the top-down influence from cognitive penetration in previous works may derive from the weaker perceptual processing priority compared with cognitive processing priority for ambiguous stimuli. According to previous work about working memory functions, contents that were absent but stored in working memory were worse in perceptual fidelity but better in cognitive parts like values and rewards [13,14,15,16]. Therefore, stimuli that are absent but stored in working memory may also lead to cognitive penetration in a way similar to that of ambiguous stimuli. Following this logical line, we hypothesized that cognitive penetration was the consequence of stronger cognitive processing over perceptual processing. Therefore, this current research aimed to examine this assumption.

We constructed experimental conditions in the realm of perception judgment based on coin value information. Following the classical paradigm in the work of Bruner and Goodman [17], who revealed that poor children have a bias to overrate coin size, the current study designed three experiments in the paradigm of perceptual judgment for coins of different values. We chose this paradigm for two reasons. Firstly, the coin size perception task was one of those classical paradigms demonstrating cognitive penetration. Manipulations of a critical independent variable, i.e., the cognitive processing priority over perception, in this paradigm would make our findings more general and robust. Secondly, the coin stimuli were reported to be more related to the cognitive control network than other stimuli such as faces, which are related to emotional pathways as well as the cognitive control network in the brain [18,19,20]. Experiment 1 was designed to disclose the fact that cognitive information from social value cognition would penetrate visual perception. It has been reported that ambiguous stimuli lead to cognitive penetration [2,7]. Experiment 1 aimed to test whether absent stimuli during perceptual decisions had the same effect of cognitive priority over perception during decision making. Specifically, participants were asked to judge the sizes of coins which were associated with different values before making a perceptual judgment. Experiment 2 aimed to assess the contribution of perception in triggering cognitive penetration. Considering previous studies highlighting the role of stimulus ambiguity in the top-down influence of perception [7,9], we hypothesized that perceptual weakness is a critical factor for cognitive penetration. In Experiment 2, participants went through a reproducing task where they were asked to adjust the size of a circle to reproduce the size of a coin simultaneously presented with the adjusted circle. According to our hypothesis, no significant difference between the adjusted radius for the circle associated with a high or low value should be observed. Experiment 3 was designed to trigger cognitive penetration again by withdrawing the perceptual input when making size judgment. We expected to observe a significant difference between circles associated with different values under the condition of poor perceptual input.

## 2. Experiment 1

Experiment 1 was designed to test whether prior knowledge of value would exert influence on size perception. According to the hypothesis of cognitive penetration, we expected that people would perceive visual objects binding with high values to be larger in size.

### 2.1. Materials and Methods

#### 2.1.1. Participants

The sample size in this experiment was set by G*Power 3.1 to be no less than twenty-three participants, with the effect size of 0.80 and the expected statistical power of 0.95, in a two-tailed, one-sample *t* test. Thirty college students from South China Normal University were recruited in this experiment. All participants gave written informed consent prior to the experiment and reported normal or corrected-to-normal vision and no history of neurological or psychiatric disorders.

#### 2.1.2. Experimental Design

Experiment 1 was a one-factor within-subject design. The independent variable was the position of the coin with larger size or higher value containing two levels: right versus left. Participants had to indicate whether the right coin or the left coin was higher in value when cued by the word “value”, or larger in size when cued by the word “size”. Since this experiment focused on size judgment influenced by value cognition, the dependent variable was the percentage of correctly choosing the coin with larger size and the reaction time (RT) for each participant.

#### 2.1.3. Experimental Stimuli

**Figure 1:**
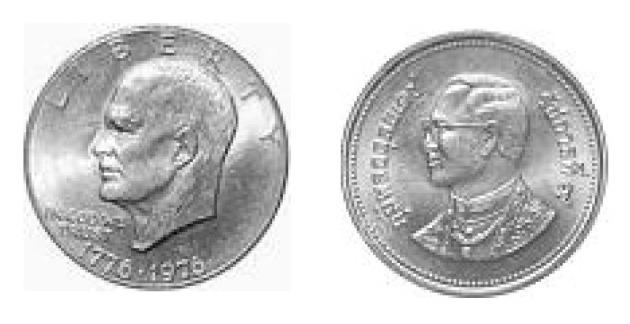
The coins used in Experiment 1. The left picture was the back side of one dollar, and the right picture was the back side of one yen. Color should be used for this figure in print.

The stimuli were pictures of American one dollar coin and Japanese one yen coin. Figure 1 displays the coins used in Experiment 1. Only the back sides of the coins were displayed because (1) the number side of the coins might induce extra influence from prior knowledge; (2) the portraits on the back side of the two coins were identical, controlling the influence of visual features. The radius of the coins was randomly chosen from 100 pixels, 110 pixels, 120 pixels, 130 pixels, 140 pixels, or 150 pixels. In each trial, two coins were centered at a 5.7° visual angle to the left and right of a fixation.

#### 2.1.4. Procedure

**Figure 2:**
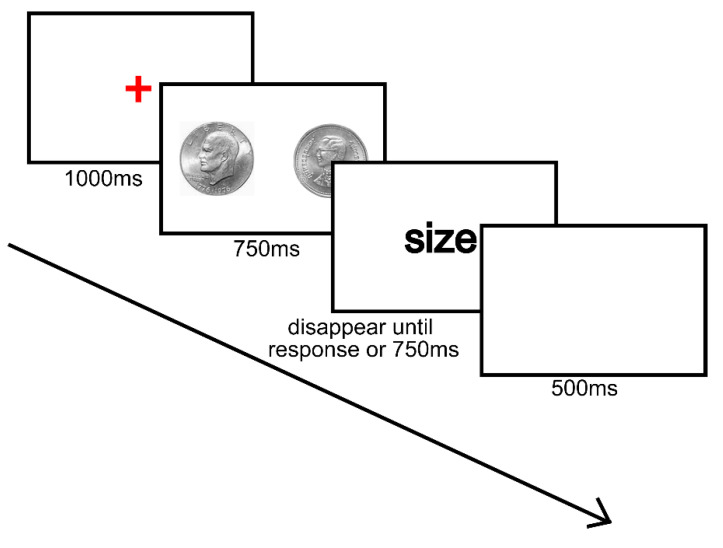
Flow graph of Experiment 1. The actual size of two coins was the same. In this example trial, participants were asked to select the physically larger coin. Color should be used for this figure in print.

All experiments were programmed using Psychtoolbox in MATLAB 2018b. Participants performed the task in individual booths, where they sat approximately 50 cm from the computer screen. Prior to the experiment, participants were explicitly informed and learned which coin portrait was higher in value. The experiment procedure of Experiment 1 is displayed in Figure 2. Each trial began with a red fixation for 1000 ms, followed by the display of two coins for 750 ms. After the disappearance of the coins, a cue word, either “size” or “value”, was presented on the screen to probe the participant to respond to the coins by pressing the key ‘J’ or ‘F’. Pressing ‘J’ indicated that the coin on the right side was larger in “size” or “value”. Pressing ‘F’ indicated that the coin on the left side was larger in “size” or “value”.

There were 520 trials in total, including 300 size-judgment trials and 220 value-judgment trials. To prevent participants from feeling bored during the task, we set the size difference between the two coins to be obvious for 80 of the 300 size-judgment trials. For the rest of the 220 size-judgment trials, there was no actual size difference so that the chance level for size judgment, without the influence of prior value knowledge, was supposed to be 0.5.

### 2.2. Results

**Figure 3:**
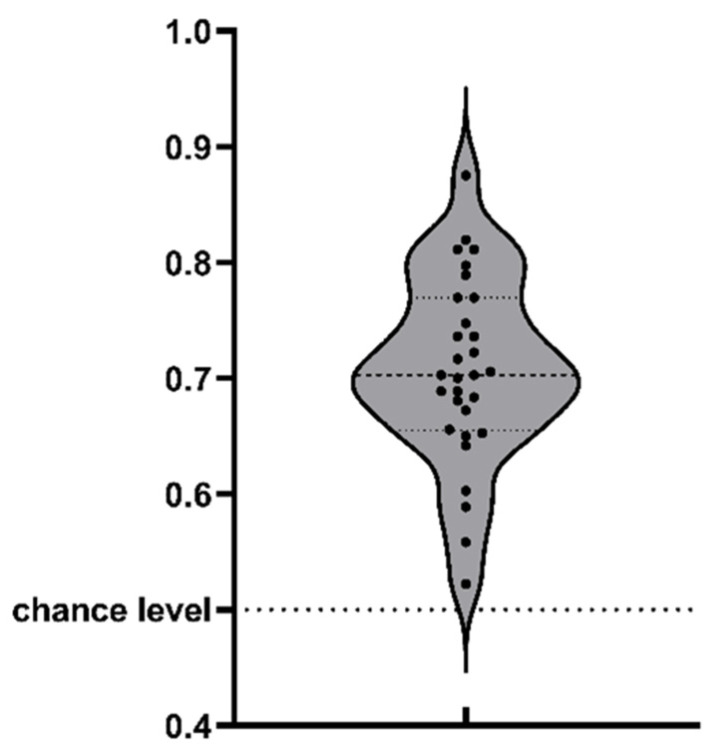
Violin plot for Experiment 1. Each dot on the plot indicates the proportion of one participant choosing one dollar coin as larger in size when the actual sizes were the same. The three dot lines above the violin plot indicate the first quartile (the bottom one), median (the middle one), and third quartile (the upper one) of the data respectively.

The averaged reaction time for all participants was 419.04 ms, with standard deviation of 96.69. The reaction times of all participants were within the range of three standard deviations from the averaged reaction time. Therefore, we did not rule out any participant based on reaction time. The data analysis was focused on size-judgment trials where the two coins were the same size. If cognitive penetration did not occur, participants were expected to be unable discern the two coins. Figure 3 displays the descriptive statistics for all data in Experiment 1. All artworks for results were programmed by the software GraphPad Prism 9. Repeated-measure ANOVA for the two factors, the coin position occupied by one dollar (left versus right) × task type (size versus value judgment), suggested significant interaction between the two factors (*F* (1, 29) = 9.523, *p* = 0.004, η^2^ = 0.052). The main effect of coin position was not significant (*F* (1, 29) = 0.090, *p* = 0.767, η^2^ = 0.0002). The main effect of task type was significant (*F* (1, 29) = 5.075, *p* = 0.032, η^2^ = 0.106). The reaction time difference between size- and value-judgment tasks was 20.069 ms (*t* = 2.253, *p* = 0.032), suggesting that choosing the higher-value one dollar cost an extra 20.069 ms in the size-judgment task than in the value-judgment task. This proved that participants’ perceptual judgment for physical size was susceptible to value information.

## 3. Experiment 2

Experiment 1 proved cognitive penetration by biased size judgment when participants lacked perceptual input. Following Experiment 1, Experiment 2 supplemented the visual perceptual input when making size judgment. Experiment two was designed to illustrate that weaker perceptual processing was a critical factor to trigger cognitive penetration. It was hypothesized that the cognitive penetration observed in Experiment 1 would disappear once the visual perceptual input was supplemented in the judgment.

### 3.1. Materials and Methods

#### 3.1.1. Participants

The sample size in this experiment was set by G*Power 3.1 to be no less than twenty-two participants, with the effect size of 0.40 and the expected statistical power of 0.95, in a repeated-measure F test. Twenty-nine undergraduate students from South China Normal University were recruited in this experiment. All participants gave written informed consent prior to participating, reported normal or corrected-to-normal vision, and reported no history of neurological or psychiatric disorders.

#### 3.1.2. Experimental Design

The experiment had two within-subject variables: the physical size of the coin (large, medium and small) and the value of the coin (high and low). The dependent variable was the radius difference between the reproduced circle and the actual coin.

#### 3.1.3. Experimental Stimuli

The coins used in Experiment 2 were the same as those used in Experiment 1, i.e., the back side of one dollar for high value and the back of one yen for low value. The large, medium, and small physical sizes were set as 70 pixels, 60 pixels and 50 pixels in radius, respectively.

#### 3.1.4. Procedure

**Figure 4:**
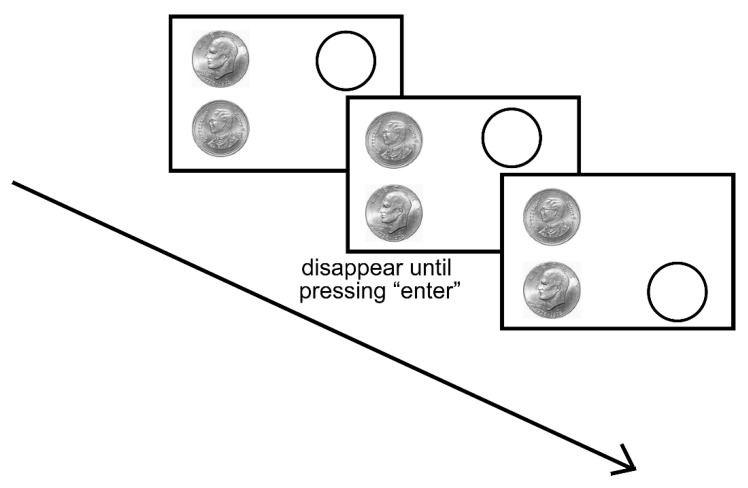
Procedure of Experiment 2. Participants were asked to adjust the radius of the circle until its radius was seen as the same as the radius of the corresponding coin. Color should be used for this figure in print.

In each trial, two coins were presented on the left half of the screen simultaneously and one circle appeared on the right half of the screen, aligning with either of the coins. Participants were asked to adjust the radius of the circle by pressing the direction keys of “↑” and “↓” on the keyboard to make the circle the same size as the corresponding coin. The key “↑” increased the radius of the circle, and the key “↓” decreased the radius of the circle. The radius of the reproduced circles was recorded. Figure 4 displays the screen for each trial presented to participants during the experiment. The relative locations of the two coins were counterbalanced between participants. The adjusted coin was randomly selected for each trial. Participants had to complete a total number of 348 trials.

### 3.2. Results

**Figure 5:**
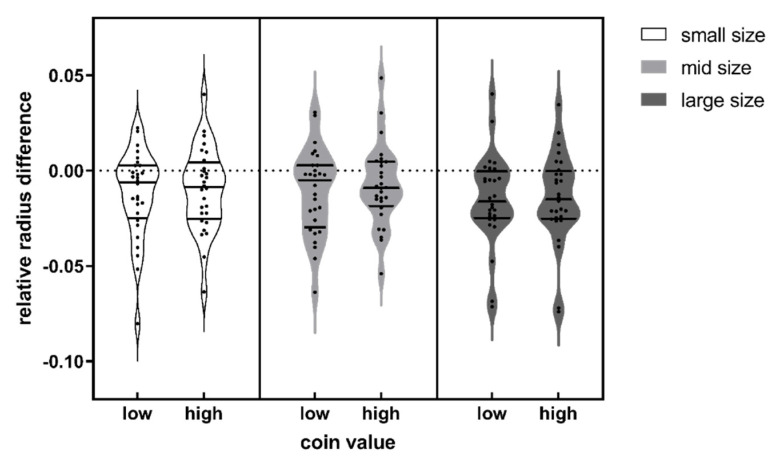
Relative radius difference between the reproduced circle and the actual coins of different sizes and values. The three solid lines above each violin plot indicate the first quartile (the bottom one), median (the middle one), and third quartile (the upper one) of the data respectively.

The descriptive statistics for data in Experiment 2 are displayed in Figure 5. We conducted repeated-measure ANOVA for the relative radius difference between the reproduced circle and the actual coin. The values of relative radius were calculated as the radius difference between the reproduced circle and the actual coin divided by the actual radius of the coin. It turned out that there was no significant interaction effect between the value and physical size (*F* (1, 27) = 1.457, *p* = 0.238). Neither the main effect for value (*F* (1, 27) = 0.947, *p* = 0.339) nor the main effect for physical size (*F* (1, 27) = 0.575, *p* = 0.455) was significant. This result possibly implies that cognitive penetration did not occur in the situation of implementing perceptual input when making size judgment.

## 4. Experiment 3

From Experiment 1 and Experiment 2, it can be inferred that weaker perceptual processing may be necessary to trigger cognitive penetration. However, it is necessary to test whether cognitive penetration would be triggered when explicitly enhancing cognitive processing to provide direct supporting evidence for our proposal. Therefore, Experiment 3 was designed to enlarge the processing difference between perception and cognition to offer direct evidence for the hypothesis that priority in cognitive processing over perceptual processing is necessary to trigger cognitive penetration. In Experiment 2, we weakened the perceptual processing and strengthened the cognitive processing by segregating the sequential display of circles and coins via masking stimuli. In this case, the time interval between perceptual input and size judgment was longer than that between cognitive processing and size judgment.

### 4.1. Materials and Methods

#### 4.1.1. Participants

The sample size in this experiment was set by G*Power 3.1 to be no less than twenty-three participants, with the effect size of 0.80 and the expected statistical power of 0.95, in a two-tailed paired *t* test. Twenty-nine college students from South China Normal University were recruited in this experiment. All participants gave written informed consent prior to participating and reported normal or corrected-to-normal vision and no history of neurological or psychiatric disorders. After the experiment, each participant would receive monetary compensation.

#### 4.1.2. Experimental Design

This was a one-factor within-subject experiment. The independent variable was the congruence between the size and value of the stimuli, including the congruent and incongruent conditions. Under the congruent condition, the circle binding with high value was physically larger than the circle binding with low value, and the circle binding with low value was physically smaller than the circle binding with high value. Under the incongruent condition, the circle binding with high value was physically smaller than the circle binding with low value, and the circle binding with low value was physically larger than the circle binding with high value. The dependent variable was the accuracy and reaction time in the size-judgment task.

#### 4.1.3. Experimental Stimuli

**Figure 6:**
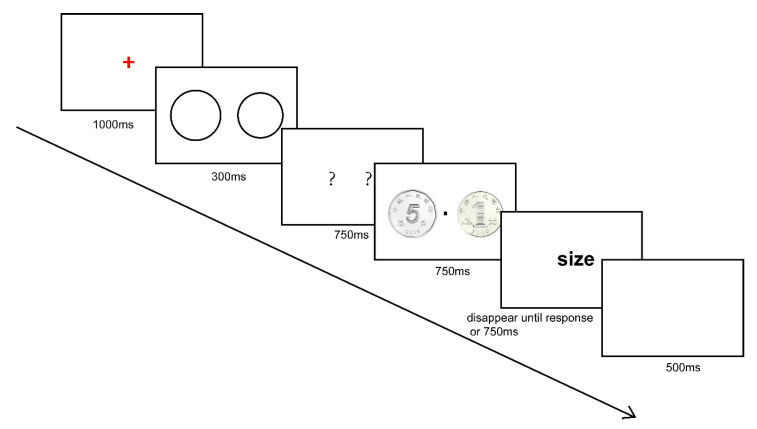
Flow graph of Experiment 3. This is an example of a congruent trial where the higher-value coin was placed in the position of the larger circle. Each trial started with a red fixation for 1000 ms, followed by two hollow circles present on screen for 300 ms. After that, two question marks were present, serving as masking stimuli for 750 ms. Following the masking stimuli, two pictures of Chinese one-yuan coin and five-jiao (i.e., half one yuan) coin were respectively located on the location of the question marks for 750 ms with a central fixation dot. The non-English term in the pictures indicates the one-yuan (right) and the five-jiao (left) coins were issued by the People’s Bank of China. Color should be used for this figure in print.

Participants were first presented with two hollow circles of different radius, and then two coins with different values against a white background (RGB: 255, 255, 255). Figure 6 shows the experimental materials and procedure of Experiment 3. The two hollow circles were two pixels in linewidth and centered at a 5.7° visual angle to the left and right of fixation. The hollow circles were the same in linewidth but differed in radius. The radius of hollow circles was determined randomly from the integral numbers ranging from 90 pixels to 110 pixels. The two coins of the same size were in the positions of the previously presented circles. One of the coins was the Chinese one yuan coin and the other was the Chinese half one yuan coin. Both of the coins were 100 pixels in radius.

#### 4.1.4. Procedure

In this experiment, participants were asked to select one circle of larger size or higher value from two circles associated with high (one yuan) or low (half one yuan) monetary values, respectively. Prior to the task, participants were told that each circle would be given a value by the upcoming coin exactly placed in the corresponding position.

In each trial, a red cross fixation was displayed in the center of the screen for 1000 ms. Then, two circles of different size appeared at the left and right side for 300 ms, followed by two masking stimuli for 750 ms. After this, two coins were presented in the same position as the circles for 750 ms. Lastly, a cue word was shown for 750 ms, which instructed participants to make reactions by ‘size’ or ‘value’. The ‘size’-asked participants to select the circle of larger size by pressing the keys ‘J’ or ‘F’, and the ‘value’-asked participants to select the circle associated with higher value. Pressing ‘J’ meant that the right circle was larger in size or higher in associated value. Pressing ‘F’ implied that the left circle was. Participants were allowed to make their response from the presentation of the cue word. The cue word would disappear either after the participant pressed the key or 750 ms had passed. Followed by the cue word, a blank screen would be presented for 500 ms, implying an upcoming trial.

All participants completed 480 trials, 320 of which were size-judgment trials where the cue word ‘size’ asked participants to judge the size of the circle. The other 160 filter trials were value-judgment trials, where the cue word asked participants to judge the value of the circle. The value-judgment trials were needed to prevent participants ignoring the processing for coin values, but rather directly judge the circle size in their memory. The size difference between two circles was obvious for 80 of the 320 size-judgment (one-quarter) trials to control the difficulty level properly. The positions (right and left) of large and small circles were counterbalanced across all trials. Half of the size-judgment trials were under the congruent condition, where larger circles were associated with higher value and smaller circles with lower value. The other half of the size-judgment trials was under the incongruent condition, where larger circles were associated with lower value and smaller circles with higher value.

### 4.2. Results

**Figure 7:**
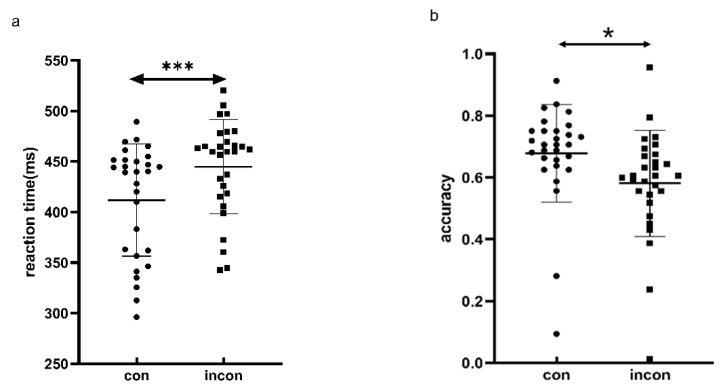
Behavioral performances of (**a**) mean reaction time; (**b**) accuracy. * indicates *p* < 0.05, and *** indicates *p* < 0.01.

We conducted a repeated-measure ANOVA on accuracy and reaction time between congruent and incongruent conditions during size and value judgments, with a significance level of 5%. Figure 7 displays all data in Experiment 3. The repeated-measure ANOVA on reaction times revealed a significant main effect of congruency (*F* (1, 28) = 36.745, *p* < 0.001, η^2^ = 0.396). The reaction time under the congruent condition was 31.825 ms faster than under the incongruent condition (*t* (28) = −6.062, *p* < 0.001), as is shown in Figure 7a. The main effect of task type was not significant (*F* (1, 28) = 0.168, *p* = 0.685, η^2^ = 0.002), nor was the interaction between congruency and task type (*F* (1, 28) = 0.315, *p* = 0.579, η^2^ = 0.0005). For accuracy, both the main effects of task type (*F* (1, 28) = 461.508, *p* < 0.001, η^2^ = 0.626) and congruency (*F* (1, 28) = 7.078, *p* = 0.013, η^2^ = 0.038) were significant. The accuracy under the congruent condition was 0.07 higher than that under the incongruent condition (*t* (28) = 2.66, *p* = 0.013), as is shown in Figure 7b. The accuracy in the size-judgment task was 0.284 lower than that in value-judgment task (*t* (28) = −21.483, *p* < 0.001). The interaction effect between congruency and task type on accuracy was not significant (*F* (1, 28) = 1.159, *p* = 0.291, η^2^ = 0.006). The result from Experiment 3 demonstrated that reaction times in size perception were affected by the monetary value of stimuli. Participants were prone to perceive high-value objects as larger.

## 5. Discussion

The reported experiments examined the influence of value on size perception. Three experiments in the paradigm of perceptual judgment for coins of different values were conducted. In Experiment 1, we observed a cognitive penetration phenomenon where participants’ size judgment was subject to value information. Specifically, participants were asked to judge the size of coins which were different in value before making a perceptual judgment, but the coins were actually the same physical size. The result suggested that participants chose the coin associated with high value as the physically larger one, which means that perceptual judgment can be influenced by cognitive processing of stimulus values, even though the stimuli are absent. Experiment 2 was designed to assess the contribution of perception in triggering cognitive penetration. Participants went through a reproducing task where they were asked to adjust the size of a circle to reproduce the size of a coin simultaneously presented with the adjusted circle. We found no significant difference between the adjusted radius for the circle associated with high or low values, suggesting that it is possible that no cognitive penetration occurred during this situation. This suggested that when visual input was present during the size judgment, the perceptual judgment would be immune to valuable information. Experiment 3 was designed to trigger cognitive penetration again by segregating the perceptual input and cognitive input to enlarge the processing difference between perception and cognition. The results suggested that cognitive penetration came back after the perceptual input was not presented. With three experiments, we proved that the processing priority of cognition over perception was a necessary trigger for cognitive penetration.

Our findings contain two aspects. Firstly, the results suggest that value information associated with an object impacted the size judgment of that object. There are several plausible perspectives to explain this phenomenon. From the perspective of cognitive control, the top-down modulation of cognition on perception has been empirically evident for a long time [1,7]. According to Bar’s model, ambiguous information from visual input may be interpreted unconsciously as information matched with representations in memory, which reveals how top-down factors can influence early visual processing. From the perspective of prediction error minimization theory [4], perception and cognition are continuous, which contributes to pattern matching between incoming external stimuli and internal mental representations. Therefore, information from the external surroundings should be extracted in a way that is consistent with predictions. From the perspective of cognitive neuroscience, a recent study, which suggested a neural basis for cognitive penetration, proved that the visual cortex encodes value information associated with objects. This study pointed out that representations within the visual area were alterable with associated values [21]. In addition, it was reported that a node of the executive attentional network involving the anterior cingulate cortex (ACC) was closely connected to sensory systems based on dynamic bottom-up and top-down interactions [22]. The attention network was recorded to be frequently activated by conflicts between stimulus attributes [23]. Another neuroscientific investigation presented participants with arrays of moving dots whose direction of motion was difficult to discern. Participants were required to report the predominant motion direction, before which participants learned that they would receive a reward if the dots predominantly moved in a particular direction. The results suggested that people tended to see the dots moving in the direction associated with reward, and did so by biasing their visual search in favor of the desired perception. Moreover, occipital regions involved in perceptual encoding and prefrontal regions involved in top-down control were modulated by these motivational effects on vision [24]. Combined with the findings of the current study, it can be inferred that value might influence size perception via the connections between the ACC and sensory system of humans, providing evidence for the proposal that factors of internal state-like values could modulate brain mechanisms through connection pathways. Further research is required for this demonstration.

Secondly, but more importantly, the cognitive processing advantage is a necessary condition for the occurrence of cognitive penetration. In our study, the cognitive processing advantage was achieved via the absence of perceptual stimuli, which resulted in insufficient or impaired perceptual processing, when making perceptual judgments. This finding makes sense when considering the proposal that there is no definite boundary between cognition and perception. It has been proposed that conscious feelings of human beings are combinations of both top-down stored knowledge and bottom-up incoming external information [4,25]. Perception and cognition are continually interacting with each other [26]. Under this premise, the findings from the current study confirmed cognitive penetration in that the absence of visual stimuli contributed to insufficient sensory information to make judgments about the external world. In other words, insufficient perceptual input led the information coming from other systems to be indispensable, thus triggering cognitive penetration. When perceptual input was presented, the cognitive penetration disappeared, as was observed in Experiment 2 of our study. However, it must be mentioned that the current study did not involve finding the boundary condition to trigger cognitive penetration. Further investigation is needed before the boundary condition for perceptual input to trigger cognitive perception is detected. Our current findings also fuel the notion of embodied cognition by revealing that visual size illusion may result from the social value of the stimuli [27]. From the perspective of embodied cognition, a plausible explanation for our experimental findings is that judgment based on the physical size of perceptual stimuli is greatly influenced by cognitive learning of the social information contained in the stimuli.

Despite the consistency with previous findings, the current study proposed another interpretation from the perspective of cognitive resources. Top-down cognitive processing and bottom-up input are continuously competing for limited cognitive resources. When the external input was withdrawn in Experiment 1 and Experiment 3 in this study, cognitive processing took the advantage of more cognitive resources. Therefore, we proposed from this study that the critical point for the occurrence of cognitive penetration is the processing priority of cognition over perception.

Some limitations of this study are worth noting. Firstly, the insignificant results in Experiment 2 implied that cognitive penetration was likely dismissed in the situation of implementing perceptual input when making size judgment. Nevertheless, the possibility that the experimental paradigm in Experiment 2 was not suitable for testing cognitive penetration cannot be ignored. We ask for future studies to test this experimental paradigm in search of cognitive penetration. Secondly, the findings of this study were obtained from college students, but whether these findings can be generalized to broader populations, especially those whose income level is different from that of college students’, still requires further exploration. Lastly, but most importantly, according to a previous discussion about how beliefs on stimulus colors influence how people perceive the color [28], there is a distinction between cognitive penetration and perceptual decision when stimuli are absent. Thus, rigorously speaking, Experiment 1 did not prove that cognitive penetration occurs, but that perceptual decision is influenced by cognitive processing regarding valuable coins.

## 6. Conclusions

The major finding that emerged from this work is that cognition priority over perception during the competition for processing resources is an indispensable condition for the occurrence of cognitive penetration. People are prone to overestimate the size of an object which is associated with higher value, compared with one with lower value, on the premise of insufficient or absent perceptual input.

## Data Availability

Raw data and analysis scripts can be found via the following link: https://osf.io/vup6w/ (accessed on 22 June 2023).
